# An Integrated Hardware–Software Platform for Automated Thermodynamic Characterization of Gas–Solid Interfaces Using a Resonant Microcantilever

**DOI:** 10.3390/mi17040428

**Published:** 2026-03-31

**Authors:** Chunfeng Luo, Haitao Yu, Naidong Wang, Fan Long, Hua Hong, Weijie Zhou, Chang Chen

**Affiliations:** 1 School of Microelectronics, Shanghai University, Shanghai 200444, China; darkcats@shu.edu.cn; 2 Xiamen High-End MEMS Technology Co., Ltd., Xiamen 361001, China; yht@highend-mems.com (H.Y.); fan.long@highend-mems.com (F.L.); hua.hong@highend-mems.com (H.H.); 3 Shanghai Lietuo Technology Co., Ltd., Shanghai 201821, China; weijie.zhou@leto-ic.com; 4 Institute of Medical Chips, Ruijin Hospital, Shanghai Jiao Tong University School of Medicine, Shanghai 200025, China

**Keywords:** resonant microcantilever, gas–solid interface, thermodynamic parameter extraction, CO_2_ adsorption, Sips model, LabVIEW automation, high-precision measurement

## Abstract

Measurement of material thermodynamic parameters plays a crucial role in understanding the interactions between host materials and guest species. Therefore, developing a general-purpose system for thermodynamic parameter measurement is of great significance. In this work, a complete gas–solid interface thermodynamic parameter measurement platform was developed based on isothermal adsorption and a resonant microcantilever testing platform. Unlike conventional adsorption measurement systems that rely on manual, multi-cycle adsorption–desorption processes, the proposed platform integrates an automated hardware–software architecture together with a stepwise concentration-gradient protocol and on-chip thermal desorption, enabling continuous and efficient acquisition of adsorption isotherms. The study includes: (i) construction of an improved thermodynamic parameter extraction model based on the Sips model, (ii) development of an integrated resonant microcantilever control and acquisition module using a modified Fourier algorithm, and (iii) implementation of an automated testing and data analysis software framework developed in LabVIEW based on the Queued Message Handler (QMH) architecture. The system was validated from both hardware performance and material testing perspectives using CO_2_ adsorption on H-SSZ-13 as a representative case. The results show that the system achieves a maximum sampling rate of 10,000 pts (points per second), with minimum root-mean-square (RMS) noise levels of 0.0083 Hz for frequency and 0.0109 °C for temperature. The PID temperature-control settling time (0.1%) is 24.9 ms, and the frequency-response settling time (0.01%) is 9.6 ms. Thermodynamic parameters including entropy change (*ΔS*), enthalpy change (*ΔH*), and Gibbs free energy change (*ΔG*) were successfully extracted during CO_2_ adsorption at 294.15 K under different relative uptakes. Reproducibility was verified across three independent samples, yielding a standard deviation of 9.1 J·mol^−1^ for *ΔS* at 2% relative uptake and relative standard deviations of 6.85% and 8.12% for *ΔH* and *ΔG*, respectively. These results demonstrate that the proposed thermodynamic measurement platform features a simple architecture, superior performance, and high reproducibility in gas–solid interface thermodynamic studies, showing strong potential for future commercialization.

## 1. Introduction

Measurement of thermodynamic parameters is an essential component in understanding material–guest interactions and guiding rational material design. Specifically, isothermal adsorption data can be used to calculate the isosteric heat of adsorption (*q_st_* or *ΔH*), which reveals the strength and nature of adsorption sites, while *ΔG* and *ΔS* characterize the spontaneity and ordering of the adsorption process. Traditionally, these quantities are obtained from multi-temperature isotherms using the Clausius–Clapeyron (C–C) relation or more robust methods such as QSDFT or isotherm fitting, and have been widely applied for screening and mechanistic analysis of porous materials [1]. Commercial adsorption instruments, such as volumetric adsorption analyzers, gravimetric microbalances, and spectroscopic adsorption systems, have become the standard tools for obtaining adsorption isotherms and derived thermodynamic parameters. These techniques provide high accuracy in equilibrium uptake measurements and benefit from well-established calibration protocols and standardized data analysis procedures. As a result, they are widely regarded as reliable benchmarks for adsorption thermodynamics characterization. However, such conventional methods also exhibit inherent limitations. They typically rely on bulk sample quantities and macroscopic averaging, making them insensitive to local adsorption heterogeneity and interfacial dynamics. In addition, thermodynamic parameters are usually extracted from discrete equilibrium points across multiple temperatures, leading to long measurement times and increased experimental complexity. The requirement for repeated adsorption–desorption cycles further amplifies systematic errors and limits throughput, while the lack of real-time, in situ capability restricts their applicability in dynamic or transient adsorption studies.

Microcantilevers are among the most representative devices in microelectromechanical systems (MEMS), with extensive applications in sensing [2,3], imaging [4,5,6], optical modulation [7], and biomedical detection [6,7,8]. As one of their milestone applications, microcantilevers serve as the core actuator and readout component in atomic force microscopy (AFM). In 1986, Binnig, Quate, and Gerber first proposed and experimentally demonstrated the AFM principle, making nanoscale surface imaging possible [9].

In recent years, resonant microcantilevers—a specialized type of microcantilever—have attracted increasing attention due to their capability to monitor nanoscale mass variations by tracking changes in resonance frequency. They have been successfully extended to thermal analysis and adsorption studies. For instance, Yao et al. proposed a MEMS-based thermogravimetric analysis (MEMS-TGA) platform capable of rapid temperature ramping and highly sensitive mass characterization using trace amounts of samples [10]. Earlier, in 2014, Xu et al. introduced the concept of “Microgravimetric Thermodynamic Modeling,” which employs resonance frequency shifts to analyze the thermodynamics and optimize the performance of gas–solid systems [11]. Subsequently, in 2016, they further proposed a fast method for extracting the activation energy (*Ea*) of molecular adsorption using resonant microcantilever analysis [12].

Recently, the application of resonant microcantilevers for thermodynamic parameter measurements has emerged as a promising direction. Zhang et al. fabricated an aminated MIL-101(Cr)-based cantilever sensor and, in combination with Xu’s microgravimetric modeling approach, extracted *ΔH*, *ΔG*, *ΔS*, and *Ea*, demonstrating the feasibility of performing adsorption thermodynamic characterization on miniaturized platforms with minimal sample quantities and rapid test cycles [13].

However, despite these advances, current thermodynamic analyses based on resonant microcantilever modeling still face several limitations. First, converting frequency shifts directly into “absolute mass” is highly sensitive to sample placement and fabrication variations, leading to inconsistent mass–frequency coefficients and poor reproducibility among devices. Second, many existing systems rely on frequent manual switching between adsorption and desorption processes, which increases testing time and introduces human error. Third, the overall system integration remains limited, and data processing often requires manual intervention, restricting scalability and industrial applicability.

To address these challenges, the present study proposes improvements from both modeling and system design perspectives. In terms of modeling, the isosteric method is rederived by replacing “absolute uptake” with “relative uptake”, thereby eliminating systematic errors caused by sample positioning and device variability. A stepwise concentration-gradient protocol is adopted instead of repetitive adsorption–desorption cycles, and on-chip heating is employed to enable rapid desorption at a single test temperature, allowing fast and continuous measurement of one complete isotherm. In terms of system design, the control and acquisition circuits of the resonant microcantilever are highly integrated onto a single PCB. An optimized Fourier algorithm is implemented to perform real-time frequency extraction on resource-limited MCUs, while an automated multi-threaded control and data analysis framework is realized in LabVIEW based on the Queued Message Handler (QMH) architecture.

In contrast to conventional systems that rely on manual intervention and repetitive adsorption–desorption cycles, the proposed approach integrates automated system control with a stepwise gradient protocol and on-chip desorption, enabling continuous, rapid, and reproducible thermodynamic measurements.

Through these improvements, a rapid, low-cost, lightweight, and automated adsorption thermodynamic measurement system with excellent reproducibility has been developed. The effectiveness and reliability of the proposed system are experimentally validated using the H-SSZ-13/CO_2_ adsorption system as a representative case study.

## 2. Materials and Methods

### 2.1. Thermodynamic Parameter Extraction Modeling

Before conducting this study, we systematically reviewed existing methods for thermodynamic parameter extraction. Traditionally, adsorption thermodynamics have been determined using two main modeling approaches. The first is direct calorimetric measurement, such as microcalorimetry or differential scanning calorimetry (DSC), which determines the isosteric heat of adsorption (*ΔH*) by recording the heat released or absorbed during the adsorption process in real time. Although this approach is straightforward and offers high accuracy, it requires complex instrumentation, involves long experimental cycles, and is difficult to apply under low-coverage or trace-sample conditions. The second approach is indirect modeling based on isothermal adsorption, in which adsorption isotherms are measured at multiple temperatures. The Clausius–Clapeyron Equation or adsorption models such as Langmuir, Sips, or Toth are then applied to fit isosteric points, allowing the calculation of *ΔH*, *ΔS*, and *ΔG*. This method is more experimentally accessible and particularly suitable for screening and comparing porous materials. Consequently, it has become the most widely adopted modeling strategy in adsorption thermodynamics research.

Against this background, Yu et al. (2014) first proposed a thermodynamic modeling method based on a resonant microcantilever platform, which integrates resonance frequency variation with isothermal adsorption modeling to analyze the thermodynamic behavior of chemical sensing materials during trace gas adsorption [11]. This pioneering work established the feasibility of conducting adsorption thermodynamic studies on a resonant microcantilever platform and provided a theoretical and methodological foundation for the present research. Building upon the modeling framework proposed by Xu et al., this study introduces several improvements and extensions. On one hand, a relative-loading-based isothermal adsorption modeling approach is developed to eliminate systematic errors caused by sample positioning and device variation in absolute mass conversion. On the other hand, the Sips model is employed to fit isosteric adsorption data, enabling a more accurate representation of site heterogeneity and intermolecular interactions. Through these advancements, we established a comprehensive resonant microcantilever–based isothermal adsorption modeling framework for precise thermodynamic parameter extraction.

#### 2.1.1. Resonance Frequency Variation and Relative Uptake

For a resonant microcantilever, it can be equivalently modeled as a spring–mass system, and its resonance frequency is expressed as Equation (1):

(1) $$f_{0}=\frac{1}{2\pi }\sqrt{\frac{k}{m_{eff}}}$$

$f_{0}$ denotes the fundamental resonance frequency of the cantilever, k is the spring constant, and $m_{eff}$ represents the effective mass of the cantilever. When a chemical or physical reaction occurs on the cantilever surface, a corresponding mass change Δm can be expressed as Equation (2):

(2) $$\Delta m=\frac{k}{4\pi^{2}}\left(\frac{1}{{f_{1}}^{2}}-\frac{1}{{f_{0}}^{2}}\right)$$

**Figure 1:**
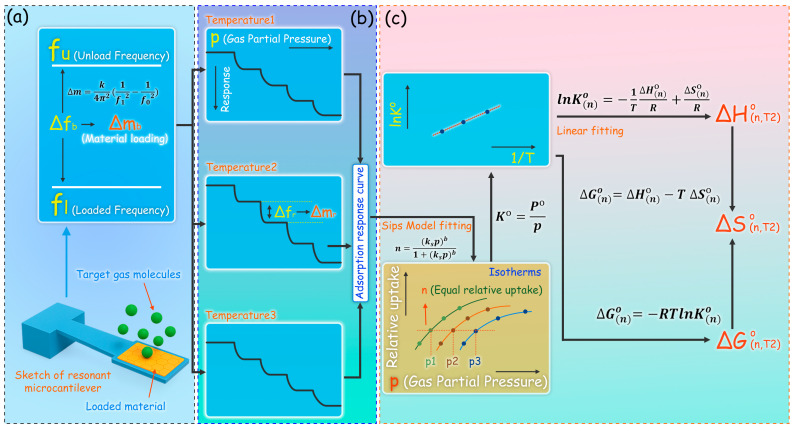
Thermodynamic parameter extraction model based on a resonant microcantilever: (**a**) Relationship between resonance frequency and relative mass change; (**b**) Stepwise concentration-gradient measurement for continuous adsorption isotherm acquisition; (**c**) Fitting of the adsorption isotherm using the Sips model and calculation of thermodynamic parameters via the Gibbs–Helmholtz Equation. Arrows indicate the data processing flow, and different colors represent distinct stages of the thermodynamic parameter extraction process.

Therefore, during the measurement of isothermal adsorption curves, as illustrated in Figure 1a, the resonance frequency of the unloaded cantilever ($\;f_{u}\;$) is first measured at the target temperature. After sample deposition, the resonance frequency becomes $f_{l}$. The corresponding sample loading mass ($\Delta m_{b}$) can then be calculated using Equation (2). As shown in Figure 1b, when a test gas with a gradually increasing concentration (or partial pressure) is introduced at a fixed temperature, the resonance frequency variation ($\Delta f_{r}$) at each gas pressure can be obtained. Using Equation (2), the adsorbed gas mass ($\Delta m_{r}$) under the corresponding temperature and partial pressure can be calculated. It should be noted that in all calculations of Δm, the spring constant k remains an unknown parameter. However, if the absolute adsorbed mass is replaced by the relative uptake, this unknown factor can be effectively eliminated, as expressed in Equation (3):

(3) $$n=\frac{\Delta m_{r}}{\Delta m_{b}}$$

It should be emphasized that the spring constant k and other proportional factors (e.g., $4\pi^{2}$) are fully eliminated in the definition of relative uptake, along with the associated errors in k caused by variations in sample deposition position and coating thickness. Therefore, no calibration of k or absolute mass is required in this method. In Equation (3), n represents the relative uptake, which can be interpreted as the ratio of the adsorbed gas mass to the mass of the loaded material. In this study, the commonly used unit of adsorption amount, mmol·g^−1^, was not adopted. This is because the proposed modeling framework is based on the conversion relationship between resonance frequency shift and mass change. Accordingly, the adsorption amount is expressed as the ratio of gas mass to sample mass. After normalization, a dimensionless relative uptake (%) is obtained. Therefore, in the isosteric analysis presented in this work, relative uptake (%) is used as the parameter for the adsorption isotherm.

#### 2.1.2. Adsorption Isotherm Fitting and Thermodynamic Parameter Extraction

After three repeated measurements, the adsorption data were fitted using the Sips isotherm model. The resulting adsorption isotherms, plotted as functions of relative uptake, are shown in Figure 1c.

**Figure 2:**
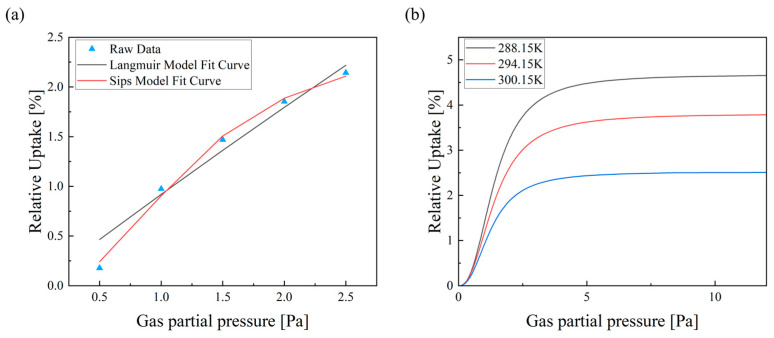
(**a**) Comparison of Langmuir and Sips model fitting curves; (**b**) Sips-model–fitted adsorption isotherms of the same H-SSZ-13 sample at different temperatures.

Regarding the choice of adsorption model, the Langmuir model describes adsorption on uniform energy sites without intermolecular interactions, whereas the Sips model accounts for heterogeneous adsorption sites and includes the effects of intermolecular interactions. When the parameter k in the Sips model equals 1, the model simplifies to the Langmuir form. As shown in Figure 2a, for the H-SSZ-13 measurements in this study, the Sips model provides a significantly better fit to the experimental data than the Langmuir model. The Langmuir fitting appears nearly linear, which is attributed to the fact that the adsorption isotherms were measured at ppm-level gas partial pressures. In the Langmuir Equation:

(4) $$\theta =\frac{k_{L}p}{1+k_{L}p}$$

when p is small and $k_{L}p\;\ll \;1$, the surface coverage θ approximates $k_{L}p$, leading to a linear relationship between θ and p. Furthermore, as shown in the Sips-model fittings of the same H-SSZ-13 sample at different temperatures, the maximum relative uptake clearly decreases with increasing temperature, which is fully consistent with adsorption thermodynamics theory.

The Sips model employed in this study is expressed as follows:

(5) $$n=\frac{{\left(k_{s}p\right)}^{b}}{1+{\left(k_{s}p\right)}^{b}}$$

In this model, n represents the relative uptake. In conventional adsorption models, n is typically defined as surface coverage or absolute adsorption amount. However, in this work, the relative uptake is used instead. Since it is defined relative to the specific sample deposited on the cantilever, it provides a more meaningful and reliable parameter under small sample mass conditions, offering lower measurement uncertainty and improved reproducibility compared with conventional coverage or absolute uptake. The parameters b and $k_{s}$ are fitting constants of the model. According to the discussion by Chen, T. [14], it is questionable to directly use experimentally fitted adsorption constants as equilibrium constants in thermodynamic calculations. Chen emphasized that adsorption equilibrium constants must be converted to dimensionless quantities under standard-state conditions. Therefore, in this work, $k_{s}$ is treated purely as a fitting parameter without direct physical meaning.

The calculation of the standard adsorption equilibrium constant ($K^{\circ }$) follows the method proposed by Myers, A. L. [15]. Myers developed a thermodynamic model (Equation 53 in his paper) for determining enthalpy (*ΔH*), entropy (*ΔS*), and Gibbs free energy (*ΔG*) changes per mole of gas adsorbed per unit mass of adsorbent. In his formulation, $f_{i}$ and $f_{\;i}^{\circ }$ are expressed in terms of the gas partial pressure (p) and the standard pressure ($P^{\circ }$). Accordingly, in this study, the standard adsorption equilibrium constant $K^{\circ }$ can be expressed as follows:

(6) $$K^{\circ }=\frac{P^{\circ }}{p}$$

Based on the Gibbs–van ’t Hoff and Gibbs–Helmholtz Equations, the following relations can be obtained:

(7) $$\Delta G_{\left(n\right)}^{\circ }=-RTlnK_{\left(n\right)}^{\circ }$$

(8) $$\Delta G_{\left(n\right)}^{\circ }=\Delta H_{\left(n\right)}^{\circ }-T\Delta S_{\left(n\right)}^{\circ }$$

(9) $$lnK_{\left(n\right)}^{\circ }=-\frac{1}{T}\frac{\Delta H_{\left(n\right)}^{\circ }}{R}+\frac{\Delta S_{\left(n\right)}^{\circ }}{R}$$

$\Delta G_{\left(n\right)}^{\circ }$ denotes the Gibbs free energy change, R is the universal gas constant, T is the absolute temperature, $\Delta H_{\left(n\right)}^{\circ }$ represents the enthalpy change, and $\Delta S_{\left(n\right)}^{\circ }$ represents the entropy change. The subscript (n) indicates that these quantities correspond to a specific relative uptake n. Unlike conventional calculations, which typically require only two adsorption isotherms, the present study employs three adsorption isotherms measured at different temperatures. The middle temperature (Temperature 2 in Figure 1c) is taken as the reference. For a given relative uptake n, the equilibrium pressures obtained at the three temperatures are used to calculate the standard adsorption equilibrium constant $K^{\circ }$ according to Equation (6). As shown in Equation (9), $lnK_{\left(n\right)}^{\circ }$ exhibits a linear relationship with 1/T. Thus, by performing linear regression, the slope of the fitted line corresponds to $-\Delta H_{\left(n\right)}^{\circ }/R$, from which the enthalpy change $\Delta H_{\left(n\right)}^{\circ }$ can be determined. Subsequently, the Gibbs free energy change $\Delta G_{\left(n\right)}^{\circ }$ is obtained from Equation (7), and the entropy change $\Delta S_{\left(n\right)}^{\circ }$ is derived from Equation (8). By repeating this calculation for each relative uptake n, the corresponding values of $\Delta H_{\left(n\right)}^{\circ }$, $\Delta S_{\left(n\right)}^{\circ }$, and $\Delta G_{\left(n\right)}^{\circ }$ can be obtained, yielding the thermodynamic parameter profiles as functions of relative uptake.

### 2.2. Design of Resonant Microcantilever Control and Data Acquisition Module

The core of the resonant microcantilever testing system lies in the design of its control and data acquisition module. This module not only coordinates multiple key functions—such as resonant microcantilever actuation, signal acquisition, and temperature regulation—but also plays a crucial role in achieving high sensitivity and stability of the overall system. In general, a complete control and data acquisition module consists of two main components: hardware circuit design and firmware/algorithm design. The hardware determines the signal quality and system response speed, whereas the firmware and algorithms govern the real-time performance and computational accuracy of the system.

#### 2.2.1. Hardware Design

The core of the hardware design lies in the circuit architecture. The overall circuit design process typically involves four main steps:

1. Designing the functional block diagram of the actuation and signal acquisition modules according to the operating principles of the resonant microcantilever;
2. Selecting appropriate electronic components based on the block diagram and completing the schematic design with peripheral circuitry;
3. Performing simulation and analysis of key analog circuit sections to ensure proper operation and signal integrity;
4. Completing the printed circuit board (PCB) layout based on the schematic, followed by PCB fabrication, assembly, and soldering.

**Figure 3:**
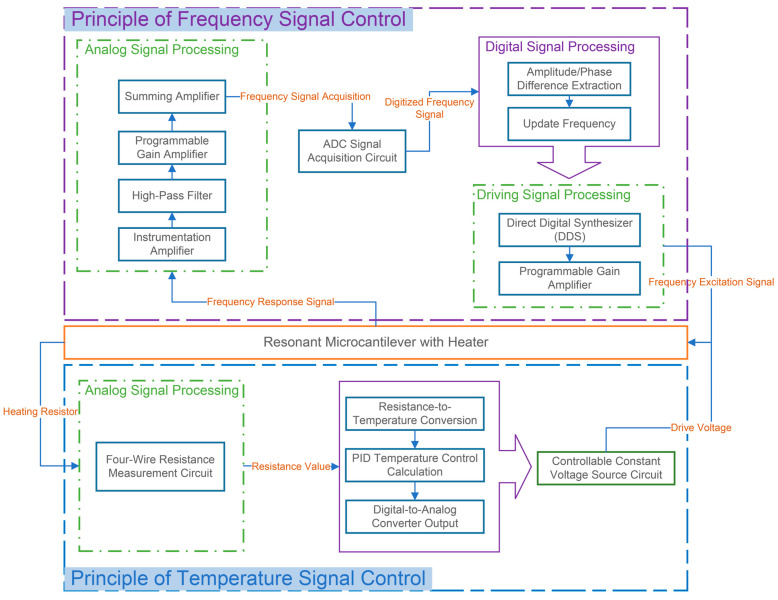
Block Diagram of the Resonant Microcantilever Driving and Data Acquisition Module with Integrated Heating Function. Arrows indicate signal flow and control paths, while different colors represent distinct functional modules within the system.

Figure 3 presents the block diagram of the resonant microcantilever driving and data acquisition module used in this study, which features an integrated on-chip heating function. The module mainly consists of two parts: the resonant signal processing section and the temperature control section. In the resonant signal processing section, the analog front-end circuitry includes an instrumentation amplifier, a high-pass filter, a programmable gain amplifier, an adder, an ADC signal acquisition circuit, and a direct digital frequency synthesizer (DDS). The algorithm layer primarily comprises the phase/amplitude extraction algorithm and the frequency update algorithm. The temperature control section contains two key analog circuits: a four-wire resistance measurement circuit and a controllable constant-voltage source circuit. Its algorithm layer includes the resistance–temperature conversion algorithm and a PID temperature control algorithm.

**Figure 4:**
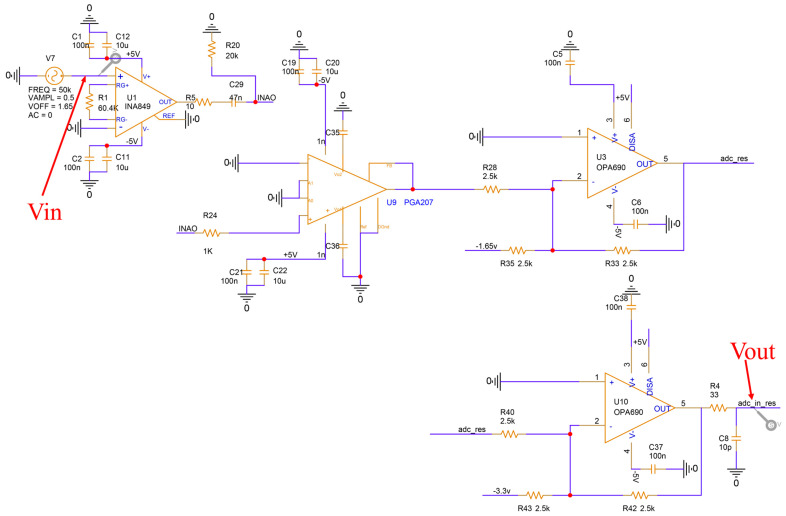
Schematic Diagram of the Resonant Microcantilever Driving Circuit Simulation.

For the resonant microcantilever driving circuit developed in this study, the most critical performance requirement is that the group delay of signal transmission must be minimal and that no waveform distortion should occur. Since the target data acquisition rate is 10,000 pts, each acquisition cycle corresponds to 100 μs. To reserve sufficient processing time for the firmware algorithms, the group delay of the circuit must be controlled within 1 μs. Therefore, PSpice was used to simulate and analyze the signal transmission characteristics of the resonant microcantilever driving circuit (Figure 4). To facilitate group delay analysis, the circuit gain was adjusted to unity by tuning the gain resistor and the programmable gain amplifier. A sinusoidal input signal $V_{in}$ with a frequency of 50 kHz, a DC offset of 1.65 V, and an amplitude of 0.5 V were applied to the circuit input, and both $V_{in}$ and $V_{out}$ waveforms were recorded.

**Figure 5:**
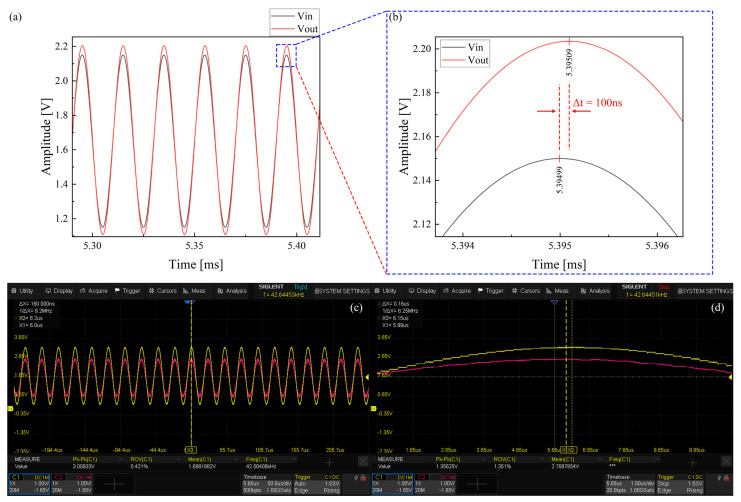
(**a**,**b**) Simulated input and output signal waveforms of the resonant microcantilever driving circuit; (**c**,**d**) experimentally measured input and output signal waveforms of the resonant microcantilever driving circuit.

As shown in Figure 5a, the simulation results indicate that the output signal $V_{out}$ exhibits no distortion, and its frequency matches that of the input signal $V_{in}$. However, the amplitude of $V_{out}$ is slightly larger than that of $V_{in}$, which deviates from the expected unity gain setting. This discrepancy arises because the INA849 (Texas Instruments, Dallas, TX, USA) instrumentation amplifier requires the external gain resistor $R_{1}$ to be disconnected (open circuit) for a gain of 1. Since PSpice does not allow unconnected pins, a 60.4 kΩ resistor was used in the schematic to approximate a unity gain, resulting in a slightly greater-than-one actual gain. As illustrated in Figure 5b, the time difference between the peaks of $V_{in}$ and $V_{out}$ is approximately 100 ns, which is far below the 1 μs threshold, indicating that the circuit’s group delay is sufficiently small and meets the design requirement. Furthermore, as shown in Figure 5c, experimental measurements on the actual circuit confirm that, when the resonant microcantilever reaches resonance, the input and output signals maintain identical frequencies with normal signal integrity. The measured delay between input and output is 160 ns, slightly higher than the simulated value. This deviation is attributed to differences between the actual parameters of the programmable gain amplifier and the INA849 used in the circuit and those assumed in the simulation. Since circuit group delay is positively correlated with open-loop gain, a higher actual gain leads to a slightly increased delay. Nevertheless, the delay remains well within the 1 μs design target.

#### 2.2.2. Firmware and Algorithm Design

Firmware serves as the bridge between hardware and software, and its design plays a critical role in ensuring the overall performance of the system. In this study, an STM32H743VIH6 (STMicroelectronics, Geneva, Switzerland) microcontroller was employed as the main control unit, responsible for data acquisition, signal processing, and logic control. In general, firmware development for embedded systems is often based on a real-time operating system (RTOS), such as FreeRTOS or RT-Thread, which facilitates task management and scheduling. However, in this work, no RTOS was used. Instead, a simpler and more efficient approach based on timers and interrupt-driven task execution was adopted. Although RTOS frameworks are well-suited for managing complex multitasking environments, their task scheduling mechanisms inevitably consume system resources, potentially degrading performance and real-time responsiveness in high-speed applications. In contrast, the firmware logic in this system is relatively straightforward. The resonant microcantilever operation involves only two stages—open-loop frequency sweeping and closed-loop frequency tracking—along with auxiliary tasks such as data transmission and reception, and communication protocol handling (packet encapsulation and parsing). Therefore, implementing an RTOS was unnecessary for this application.

**Figure 6:**
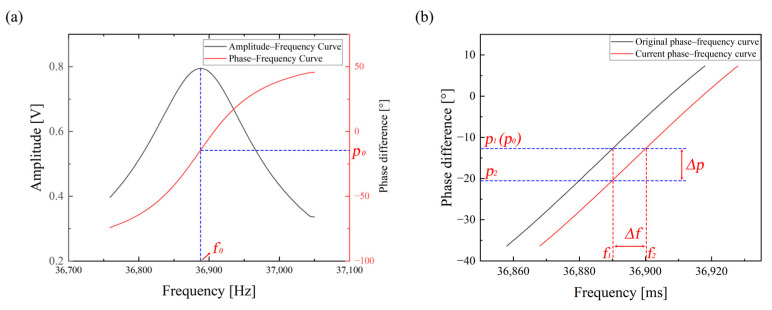
(**a**) Open-loop frequency sweep results of the resonant microcantilever; (**b**) principle of closed-loop frequency tracking.

During the open-loop frequency sweep stage, the main control unit (MCU) generates a sinusoidal signal of a fixed frequency through the direct digital synthesizer (DDS), which is controlled via the serial peripheral interface (SPI). The output signal is then amplified by an operational amplifier and applied to the driving terminal of the resonant microcantilever as the excitation (driving) signal. Simultaneously, the processed response signal from the resonant microcantilever is acquired and subjected to digital signal processing by the MCU. By comparing the excitation and response signals, both the amplitude of the response and the phase difference between the two signals at the current excitation frequency can be obtained. Figure 6 shows the experimentally measured frequency sweep of the cantilever prior to sample loading. The frequency is then incremented step by step, and this process is repeated until the complete frequency response is acquired, as illustrated in Figure 6a. For the resonant microcantilever, when the excitation frequency matches its natural resonance frequency, the response amplitude reaches its maximum. Therefore, in the amplitude–frequency characteristic curve shown in Figure 6a, the frequency corresponding to the maximum amplitude is defined as the resonance frequency ($\;f_{0}\;$), and the corresponding phase difference in the phase–frequency characteristic curve is denoted as $p_{0}$. Under ideal conditions, for a given system and resonant microcantilever, the phase corresponding to the resonance frequency ($\;f_{0}\;$) remains constant at $p_{0}$, regardless of variations in microcantilever mass or temperature. Thus, during the closed-loop tracking stage, the system continuously adjusts the excitation frequency so that the phase difference between the excitation and response signals remains equal to $p_{0}$. In this way, the real-time variation in the resonance frequency with time can be accurately tracked.

During the closed-loop tracking stage, the frequency update can be expressed as follows:

(10) $$f_{2}=f_{1}+\frac{\left(p_{2}-p_{1}\right)}{k_{p}}$$

In this Equation, $f_{2}$ and $p_{2}$ represent the current resonance frequency and phase difference, respectively, while $f_{1}$ and $p_{1}$ denote the resonance frequency and phase difference from the previous time step. Under ideal conditions, $p_{1}$ equals the reference phase $p_{0}$. The parameter $k_{p}$ represents the slope of the phase–frequency characteristic curve within the linear region centered around $p_{0}$, with a unit of degrees per hertz (°/Hz). During frequency tracking, the phase–frequency characteristic curve shifts laterally with changes in mass or temperature. Consequently, when the actual resonance frequency has shifted, the system continues to excite the resonant microcantilever at the previous frequency $f_{1}$, resulting in a new phase difference $p_{2}$. To restore the phase difference to $p_{0}$, a frequency adjustment Δf is required. The updated resonance frequency $f_{2}$ can then be calculated using Equation (10).

In the signal processing of resonant microcantilevers, the most critical task is the extraction of amplitude and phase difference. The most commonly used method for this purpose is the Fast Fourier Transform (FFT), which has a computational complexity of $O(N{log}_{2}N)$. In applications where data are processed on a personal computer (PC), FFT is highly efficient. For instance, Wang S. (2023) utilized an NI data acquisition card to collect resonant microcantilever data, which were then transmitted to a PC for processing in LabVIEW [16]. In such PC-based systems, FFT algorithm libraries are abundant, easy to use, and computational resources are not a constraint. However, transmitting data back to a host PC for FFT processing introduces significant latency and bandwidth consumption, which are unacceptable for high-speed applications such as those in the present study. On the other hand, performing FFT-based amplitude and phase extraction directly on a resource-limited microcontroller unit (MCU) can easily lead to computational bottlenecks, as the MCU must handle both signal acquisition and intensive FFT computation simultaneously. Therefore, an alternative method for extracting amplitude and phase difference is required.

In this context, Ruppert et al. (2017) proposed a signal modeling approach based on the mechanical dynamics of AFM cantilevers [17], where the cantilever vibration signal is expressed as a modulated waveform:

(11) $$z\left(t\right)=A\left(t\right)\cos\left(w_{c}t+\varphi \left(t\right)\right)$$

A(t) represents the time-varying vibration amplitude of the microcantilever, $\omega_{c}$ is the carrier angular frequency, and φ(t) denotes the instantaneous phase. The paper points out that by multiplying this signal with the reference orthogonal basis functions $cos(\omega_{c}t)$ and $sin(\omega_{c}t)$, and then performing integration (i.e., a digital inner product operation), the in-phase (Equation (12)) and quadrature (Equation (13)) components can be obtained:

(12) $$I\left(t\right)=\frac{2}{T}\int_{t-T}^{t}z\left(\tau \right)\cos\left(w_{c}\tau \right)d\tau$$

(13) $$Q\left(t\right)=\frac{2}{T}\int_{t-T}^{t}z\left(\tau \right)\sin\left(w_{c}\tau \right)d\tau$$

T denotes the integration time, t represents the current time, and z(τ) is the input signal, which is equivalent to z(t) in Equation (11).

Accordingly, the amplitude (Equation (14)) and phase (Equation (15)) can be calculated using the following expressions:

(14) $$A\left[t\right]=\sqrt{I{\left(t\right)}^{2}+Q{\left(t\right)}^{2}}$$

(15) $$\varphi \left(t\right)=arctan\frac{Q\left(t\right)}{I\left(t\right)}$$

Similarly, the same mathematical framework is presented in the white paper published by Zurich Instruments, a standard manufacturer of international lock-in detection systems [18].

Therefore, building upon the existing studies, this work proposes an improved algorithm tailored for the control of resonant microcantilevers. The sampled signal can be expressed as follows:

(16) $$x\left[n\right]=A\cos\left(\Omega n+\varphi \right)$$

x[n] represents the sampled signal sequence, n is the index of the sequence element, A denotes the signal amplitude, $\Omega =2\pi f_{0}T_{s}$ is the discrete-time angular frequency, and φ represents the phase. By performing the inner product operation, the total signal power S can be obtained as follows:

(17) $$S=\overset{N-1}{\underset{n=0}{\sum }}x\left[n\right]\times x\left[n\right]=\overset{N-1}{\underset{n=0}{\sum }}A^{2}\cos^{2}\left(\Omega n+\varphi \right)$$

N denotes the total number of samples in the signal sequence. The average power can be expressed theoretically as:

(18) $$\frac{S}{N}=\frac{A^{2}}{2}$$

Accordingly, the signal amplitude can be derived as:

(19) $$A=\sqrt{\frac{2S}{N}}$$

For phase extraction, let the excitation signal sequence be:

(20) $$d\left[n\right]=D\cos\left(\Omega n\right)$$

and the response signal sequence be:

(21) $$r\left[n\right]=R\cos\left(\Omega n+\varphi \right)$$

In Equations (20) and (21), D represents the amplitude of the excitation signal, and R represents the amplitude of the response signal. Since the excitation and response signals share the same frequency, the excitation signal can be directly used as the reference signal. By performing an inner product between the two sequences, the in-phase component can be obtained as:

(22) $$I=\overset{N-1}{\underset{n=0}{\sum }}d\left[n\right]\times r\left[n\right]$$

Subsequently, the response sequence r[n] is phase-shifted by 90° to the left, yielding the quadrature component:

(23) $$Q=\overset{N-1}{\underset{n=0}{\sum }}d\left[n\right]\times r\left[n+\Delta n\right]$$

where Δn represents the element offset corresponding to a 90° phase lead in the sampled sequence. Finally, the phase difference between the excitation and response signals can be calculated using:

(24) $$\varphi =arctan\frac{Q}{I}$$

The computational complexity of this algorithm is only O(N), which reduces the computational load by a factor of ${log}_{2}N$ compared with the FFT. Moreover, unlike FFT-based methods that require separate extraction of the excitation and response phases followed by subtraction to obtain the phase difference, the proposed algorithm directly extracts the phase difference. In addition, it eliminates the curve-fitting step involved in FFT phase extraction, thereby significantly improving data accuracy and reducing noise sensitivity. Detailed experimental results of system noise performance are provided in the Results section.

### 2.3. Software Design

In a data acquisition system, the software component apart from the firmware is generally referred to as the host computer (upper computer). The primary functions of the host computer include data retrieval, visualization, processing, and storage, as well as human–machine interaction and process control. Owing to its access to abundant computing and graphical resources, the host software can be designed with high flexibility and customizability.

1. In this study, the software (hereinafter referred to as the host application) is designed with the following major functions.
2. Data communication: receiving data in real time from the resonant microcantilever control and data acquisition module (hereinafter referred to as the embedded controller) via a USB interface, followed by data unpacking, processing, storage, and visualization.
3. Human–machine interaction: responding to user operations such as start/stop testing, parameter configuration, and mode switching.
4. Automated test control: managing the coordinated operation of all system modules and instruments, automatically starting and terminating experiments according to predefined parameters.
5. Data analysis: processing experimental data based on the thermodynamic parameter extraction model and outputting the corresponding thermodynamic parameters.

During the development of the host system, various programming environments were evaluated for their functional characteristics, ecosystem support, and applicability. C#/.NET provides excellent native support for Windows and a rich graphical user interface library, making it well-suited for industrial monitoring and instrument control systems. Python offers extensive open-source resources and scripting convenience, enabling high development efficiency in research-oriented data acquisition and analysis. C++/Qt combines high performance with cross-platform capability, ideal for industrial-grade real-time control and multithreaded applications requiring high reliability. LabVIEW, on the other hand, is a graphical programming environment equipped with comprehensive toolkits for data acquisition, signal processing, and control, particularly suitable for rapid development of automated experimental and testing systems.

Considering the specific requirements of this study—including multi-channel real-time data acquisition, automated experiment control, and synchronized communication with multiple instruments (such as gas mixing systems and frequency/temperature acquisition modules)—LabVIEW (2025 Q1, National Instruments, Austin, TX, USA) was selected as the development platform for the host computer software.

**Figure 7:**
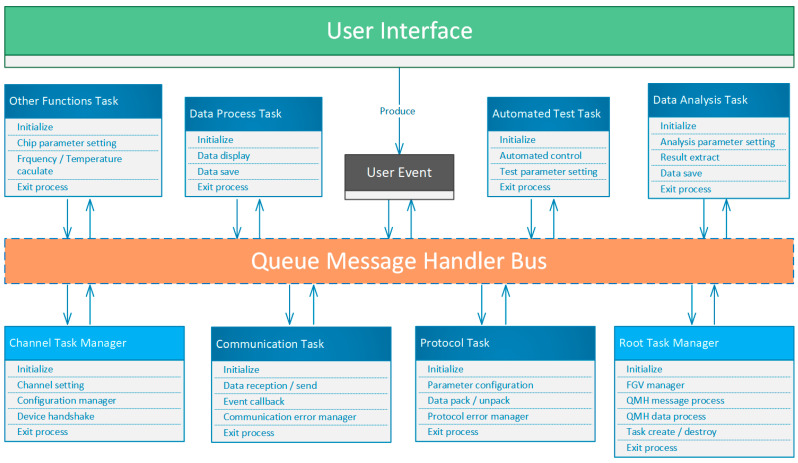
Software Architecture Based on LabVIEW and QMH. Arrows indicate the direction of control flow and message/data interaction between modules, while different colors represent distinct functional modules within the system architecture.

Figure 7 illustrates the software architecture of the host computer system developed in this study. The entire framework is built upon LabVIEW’s Queued Message Handler (QMH) architecture, which enables modular task scheduling, asynchronous event handling, and parallel processing through queue-driven message dispatching [19,20,21]. The system is organized into two hierarchical layers: the User Interface Layer and the Task Execution Layer.

At the upper level, the User Interface (UI) serves as the entry point for human–machine interaction, managing experiment initiation, control, parameter input, and real-time monitoring and visualization. User operations—such as parameter modification, test initiation, or data export—are encapsulated as User Events and broadcast to the execution layer via the QMH Message Bus. This event-driven mechanism effectively separates UI logic from task logic, ensuring high responsiveness and scalability even during multitasking operation [19,22].

The lower level, the Task Execution Layer, forms the core of the system and contains multiple functional modules and managers. All tasks are designed as reentrant tasks, allowing them to run independently within their respective channels without mutual interference [23]. The task structure within this layer can be categorized into two main types:

1. Task Manager modules, including the Root Task Manager and Channel Task Manager;
2. General Task modules, including the Communication Task, Protocol Task, Data Process Task, Automated Test Task, Data Analysis Task, and Other Functions Task [24,25].

The Root Task Manager acts as the central coordinator of the entire system—the “architect” of the software. It is responsible for system-level resource initialization and maintenance, such as managing global variables (FGVs), scheduling QMH message queues, and creating or destroying task instances. When a new channel creation request is triggered by the UI, the Root Task Manager allocates a corresponding channel instance based on task type and dispatches task instructions to the Channel Task Manager via message queues.

The Channel Task Manager serves as the “construction team,” responsible for task organization and execution. It dynamically assembles multiple General Task modules to form a complete experimental channel. For example, a typical data acquisition channel may consist of Communication, Protocol, Data Processing, and Data Saving tasks. The Channel Task Manager uses an internal message scheduling mechanism to control the execution order, synchronization, and exception handling among these modules, thereby enabling complex operations such as frequency tracking and data acquisition, temperature closed-loop control, and instrument synchronization.

The General Task modules serve as the fundamental operational units—the “building materials” of the system—and handle specific functional logic, including:

- Communication Task: Manages low-level data transmission and event callbacks, supporting multiple interfaces such as serial, TCP, or USB;
- Protocol Task: Handles data-frame encapsulation and parsing, including error checking and retransmission mechanisms;
- Data Process Task: Performs real-time data display, buffering, and file saving;
- Automated Test Task: Controls experimental workflows, programmable temperature regulation, gas-mixing adjustments, and parameter updates;
- Data Analysis Task: Performs result evaluation, curve fitting, and parameter extraction;
- Other Functions Task: Provides auxiliary functions, such as chip configuration and frequency–temperature conversion.

All tasks communicate asynchronously through the QMH Message Bus, achieving full decoupling between modules. Commands and data are exchanged exclusively through message queues, resulting in a highly modular, maintainable, and extensible architecture [19,21]. Furthermore, memory-mapping technology is introduced for efficient data buffering and file sharing, ensuring that the system maintains stable real-time performance even at high sampling rates [24].

Overall, this architecture demonstrates excellent performance in experimental automation, data acquisition throughput, and system scalability, providing a robust software foundation for future multi-channel experiment control and data analysis applications [20,25].

### 2.4. Construction of the Experimental System

**Figure 8:**
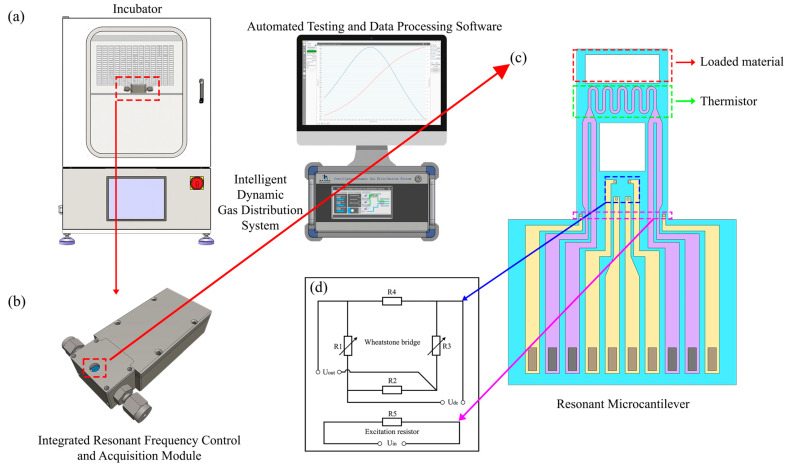
(**a**) Configuration of the thermodynamic parameter measurement system; (**b**) integrated resonant microcantilever control and data acquisition module; (**c**) resonant microcantilever with integrated heating function; (**d**) circuit diagram of the front-end driving components of the resonant microcantilever. Arrows indicate the mapping and magnified relationships from system-level components to detailed structures, while dashed boxes highlight the regions selected for zoom-in.

Based on the above studies, the thermodynamic parameter testing system developed in this work is shown in Figure 8a. The system consists of an incubator, an integrated resonant microcantilever control and data acquisition module, and an intelligent dynamic gas-mixing system. The resonant microcantilever module is placed inside the incubator and connected to the external gas-mixing system through gas lines. The host computer, equipped with the data acquisition and processing software, communicates with both the gas-mixing system and the resonant microcantilever control module via USB connections for data transmission and system control. A LoC GDS 4000 gas-mixing system (Xiamen High-End MEMS Technology Co., Ltd., Xiamen, China) was employed to accurately regulate the CO_2_/N_2_ composition and partial pressures during the measurements. The resonant microcantilever sensors used in this work were also purchased from the same manufacturer.

The incubator provides a stable and precise thermal environment for the entire experiment, minimizing temperature fluctuation effects on the results. The temperature of adsorption isotherm measurements is controlled by the external incubator, while the on-chip heating of the cantilever is used only for rapid thermal desorption between measurement cycles. The intelligent gas-mixing system enables programmable generation of target gas mixtures with precise and adjustable concentrations. The resonant microcantilever is mounted on the control and acquisition module via a socket connection, as shown in Figure 8b,c.

As illustrated in Figure 8d, the front end of the resonant microcantilever integrates an excitation resistor and a Wheatstone bridge. The excitation resistor serves as the input terminal for the external driving signal—a sinusoidal waveform with a DC bias—while one side of the Wheatstone bridge is supplied with DC power and the other serves as the output terminal. The excitation resistor periodically heats the cantilever tip in synchronization with the driving frequency, inducing cyclic thermal expansion and contraction. This oscillation causes the Wheatstone bridge to generate a sinusoidal output signal at the same frequency as the input. The thermal actuation is achieved with a typical excitation power of approximately 2–3 mW.

Overall, the entire measurement setup requires only three primary instruments and one computer, making the system simple, compact, and cost-effective to implement.

## 3. Results

In this study, an integrated resonant microcantilever control and data acquisition module was designed and developed. By combining this module with an incubator and a dynamic gas-mixing system, a complete thermodynamic parameter measurement platform was established. The system employs the equal loading method in conjunction with Sips model fitting, enabling the extraction of enthalpy (*ΔH*), entropy (*ΔS*), and Gibbs free energy (*ΔG*) under different loadings during gas adsorption at constant temperature.

To evaluate the reliability of the developed system, its performance was systematically assessed from the perspectives of hardware, firmware, and algorithm design. Subsequently, thermodynamic parameter measurements of CO_2_ adsorption on three H-SSZ-13 samples at 294.15 K (21 °C) were conducted to further verify the accuracy and reproducibility of the system. Unless otherwise specified, temperatures used in thermodynamic analysis are expressed in Kelvin (K), while degrees Celsius (°C) are used for system performance metrics such as temperature stability and noise for clarity.

### 3.1. Design and Performance of the Control and Acquisition Module

#### 3.1.1. Hardware Design Results

The resonant microcantilever control and data acquisition module developed in this study integrates all circuitry onto a compact single PCB. The on-chip temperature control and analog signal processing section consists of a four-wire resistance measurement circuit and a controllable constant-voltage source circuit. The resonant signal acquisition and analog processing section includes a direct digital signal generator (DDS), an instrumentation amplifier, a high-pass filter, a programmable-gain operational amplifier, and an adder. The power management section employs a TPS65133 power management IC, which integrates a boost converter, charge-pump inverter, and high-PSRR, high-precision LDO regulators, ensuring low noise and stable supply for the analog circuitry. The entire module was first modeled in 3D CAD software, and the enclosure was fabricated using CNC-machined aluminum alloy for mechanical strength and thermal stability.

**Figure 9:**
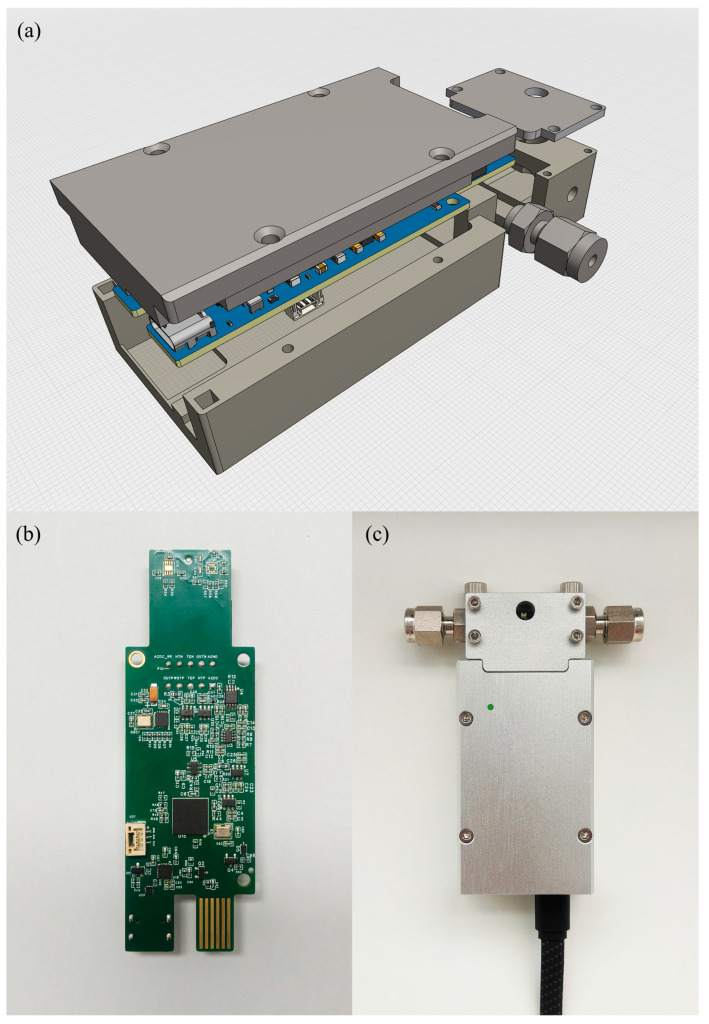
(**a**) Three-dimensional rendering of the resonant microcantilever control and data acquisition module; (**b**) photograph of the PCB layout of the resonant microcantilever control and data acquisition module; (**c**) assembled module in its final form.

The PCB measures 93 mm × 30 mm × 8 mm, while the assembled module has dimensions of 97 mm × 75 mm × 22 mm, as shown in Figure 9a,b. During operation, the module consumes approximately 1.2 W of power and supports both power supply and data communication via USB.

#### 3.1.2. Firmware and Algorithm Results

In the developed firmware, no real-time operating system (RTOS) was employed; instead, timer interrupts were directly used to trigger and schedule most of the system tasks. The frequency signal processing is implemented using a customized modified Fourier transform algorithm, which handles operations such as phase difference extraction and amplitude calculation. This algorithm reduces the computational demand to $1\;/\;{log}_{2}N$ of that required by a standard FFT and operates without relying on an FPU (Floating Point Unit). For temperature control, a PID (Proportional–Integral–Derivative) algorithm was adopted. Experimental testing demonstrated that, using an STM32H7-series microcontroller, the system achieved a data acquisition rate of 10,000 frequency–temperature points per second, with a control response delay of only 100 μs.

#### 3.1.3. Performance Characterization

**Figure 10:**
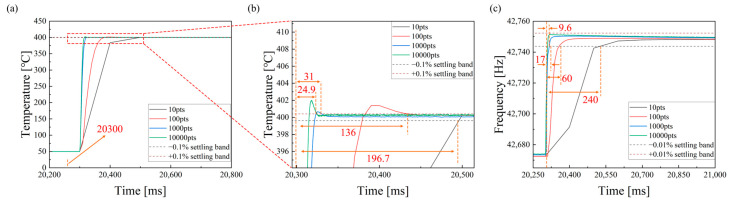
Step-response settling time test of the developed system. (**a**,**b**) Temperature response of the PID controller under a 50–400 °C step change, showing the regulation process within the ±0.1% steady-state temperature range. (**c**) Corresponding frequency response under the same step-heating condition, where the dashed lines indicate the ±0.01% steady-state frequency range.

The developed module demonstrates excellent overall stability and best-case performance across sampling rates ranging from 10 to 10,000 points per second. As shown in Figure 10 and Table 1, the PID temperature controller achieved a fastest settling time of 24.9 ms (0.1%), enabling rapid step responses up to 400 °C, while the corresponding frequency response also stabilized within 9.6 ms (0.01%). Even at lower sampling rates, the system maintained stability within 200 ms, confirming its robust control capability. In terms of precision, the module consistently exhibited ultra-low noise, with a frequency RMS noise as low as 8.3 × 10^−3^ Hz and a temperature RMS noise of approximately 1.1 × 10^−2^ °C. These results highlight not only the best-in-class sensitivity and speed of the system but also its reliable performance under all operating conditions, providing a robust foundation for high-precision and fast-response adsorption thermodynamics measurements.

### 3.2. System Validation Through Thermodynamic Measurements

#### 3.2.1. Experimental Procedure for a Single Test

**Figure 11:**
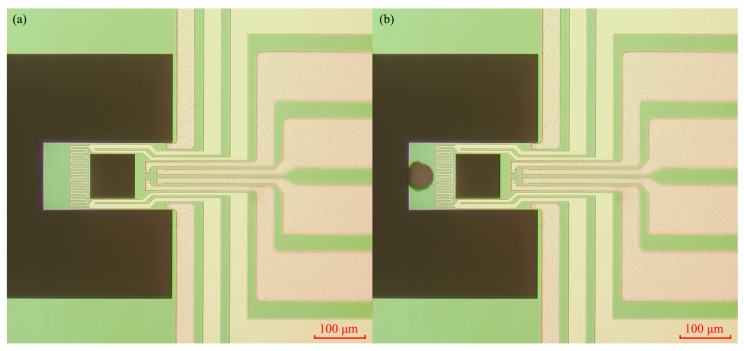
(**a**) Microscopic image of the unloaded resonant microcantilever; (**b**) microscopic image of the resonant microcantilever loaded with H-SSZ-13 sample.

To verify the functionality and reproducibility of the developed system, thermodynamic parameter measurements of CO_2_ adsorption on H-SSZ-13 were conducted. The detailed experimental procedure is as follows:

1. The uncoated resonant microcantilever was first stabilized in dry nitrogen (N_2_) at an ambient temperature of 294.15 K (21 °C), and its steady-state resonance frequency was recorded as the baseline frequency.
2. The H-SSZ-13 powder was dispersed in distilled water and deposited onto the microcantilever using a micro-spotter. The coated cantilever was then dried in an oven at 343.15 K (70 °C). The resulting morphology of the coated cantilever is shown in Figure 11.
3. The loaded cantilever was placed inside the incubator and connected to the gas-mixing system. The incubator temperature was set to 288.15 K (15 °C), and the gas-mixing system was configured to equilibrium gas conditions (N_2_) for purging.
4. The CO_2_ partial pressure sequence of the gas-mixing system was set from low to high as 5, 10, 15, 20, 25, 35, and 45 ppm (balanced with N_2_). After completing each adsorption curve, the system automatically initiated a rapid desorption process at 525.15 K (250 °C) for 1 h before proceeding to the next test. This high-temperature desorption step is enabled by the on-chip heating function of the cantilever and is necessary to ensure complete removal of adsorbed gas species between measurement cycles.
5. After completing the adsorption test at the initial temperature, the incubator temperature was adjusted to 294.15 K (21 °C) and 300.15 K (27 °C), and step (iv) was repeated to obtain adsorption data at the three temperatures.
6. Upon completion of the adsorption–desorption cycles at 288.15 K (15 °C), 294.15 K (21 °C), and 300.15 K (27 °C), the recorded frequency shift data were processed by the software to automatically extract the corresponding thermodynamic parameters (*ΔG*, *ΔH*, and *ΔS*). The selected temperature range ensures sufficient adsorption sensitivity for reliable measurement, as adsorption capacity decreases significantly at elevated temperatures. Previous studies on H-SSZ-13 provide supporting evidence for this selection (e.g., Pham et al., Langmuir, 2013 [26]), where adsorption measurements were conducted between 273 K and 343 K, overlapping with the temperature range investigated in this work.

During each adsorption stage, the system continuously tracked the resonance frequency of the microcantilever until a predefined stability criterion was met, after which it automatically switched to the next gas partial pressure. The temperature control, gas switching, and data acquisition were all automated and synchronized through the custom-developed control software.

#### 3.2.2. Thermodynamic Measurement Results

**Figure 12:**
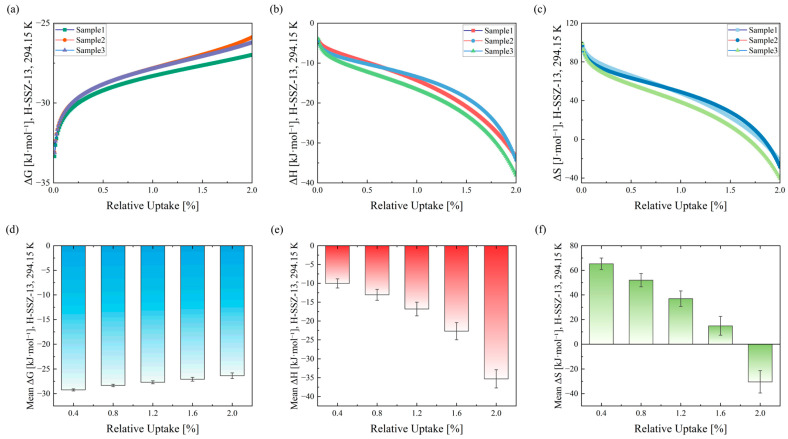
Thermodynamic parameters of CO_2_ adsorption on H-SSZ-13 at 294.15 K (21 °C). (**a**–**c**) Gibbs free energy change (*ΔG*), enthalpy change (*ΔH*), and entropy change (*ΔS*) as functions of the adsorbed amount for three independently measured samples (Sample 1–3). (**d**–**f**) Mean values of *ΔG*, *ΔH*, and *ΔS* at selected coverages (0.4%, 0.8%, 1.2%, 1.6%, and 2.0%) with error bars representing ±SD (n = 3 samples).

The developed system successfully measured the thermodynamic parameters of CO_2_ adsorption on H-SSZ-13 at 294.15 K (21 °C) under different relative uptakes. The test results obtained from three samples are presented in Figure 12 and Table 2.

As shown in Figure 12a–c, the Gibbs free energy change (*ΔG*), enthalpy change (*ΔH*), and entropy change (ΔS) exhibit consistent trends across all three samples. *ΔG* remains negative and increases in magnitude with increasing relative uptake, indicating spontaneous adsorption behavior. Both *ΔH* and *ΔS* decrease with increasing uptake; *ΔH* is consistently negative, while *ΔS* transitions from positive at low uptake to negative at high uptake. Figure 12d–f present the mean values of *ΔG*, *ΔH*, and *ΔS* at representative relative uptakes (0.4%, 0.8%, 1.2%, 1.6%, and 2.0%), with error bars representing ±SD (n = 3 samples).

Table 2 summarizes the mean values, standard deviations (SD), and relative standard deviations (RSD) of *ΔG*, *ΔH*, and *ΔS* at the same representative relative uptakes. For *ΔS*, RSD values are not reported in regions where the mean values approach zero, as they are not meaningful. The SD and RSD of *ΔG* increase with relative uptake, reaching a maximum SD of 2.141 kJ·mol^−1^ at a mean value of −26.358 kJ·mol^−1^ and an RSD of 8.123% at 2% uptake. In contrast, *ΔH* shows opposite trends in SD and RSD: SD increases while RSD decreases with uptake. At 2% relative uptake, *ΔH* reaches a maximum SD of 2.419 kJ·mol^−1^, with a mean of −35.299 kJ·mol^−1^ and an RSD of 6.853%. The SD of *ΔS* increases with uptake, reaching a maximum of 9.105 kJ·mol^−1^ at a mean of −30.396 kJ·mol^−1^ at 2% uptake.

In Figure 12, the variations in ΔG, *ΔH*, and *ΔS* during CO_2_ adsorption on the three H-SSZ-13 samples exhibit a high degree of consistency. As shown in Table 2, at a relative uptake of 2%, the RSD values of *ΔG* and *ΔH* are 8.123% and 6.853%, respectively, confirming the excellent reproducibility and stability of the developed system.

## 4. Discussion

### 4.1. Reliability of the System

The thermodynamic parameter trends obtained from the developed system are consistent with adsorption theory, confirming the reliability of the measurement results.

First, the Gibbs free energy change (*ΔG*) remains negative across the entire range of surface coverages, indicating that CO_2_ adsorption on H-SSZ-13 proceeds spontaneously under the studied conditions. This finding aligns with classical thermodynamic principles, where *ΔG* < 0 signifies a spontaneous process under isothermal and isobaric conditions [27,28,29,30].

Second, the enthalpy change (*ΔH*) is consistently negative, demonstrating that the adsorption process is exothermic. Unlike the commonly reported decrease in |*ΔH*| with increasing coverage, our results show that |*ΔH*| becomes more negative at higher uptakes. Similar trends have been observed in certain metal–organic frameworks (MOFs), where the isosteric heat of adsorption (*Q_st_*) increases with CO_2_ loading due to enhanced guest–guest interactions and stronger framework binding. For instance, Wasik et al. reported that in the M-MOF-74 series, *Q_st_* increases with CO_2_ uptake as a result of stronger CO_2_ binding at higher coverages [31]; Qazvini et al. found that in MUF-16, the increase in *Q_st_* at high uptakes originates from attractive intermolecular interactions [32]; and Ribeiro et al. demonstrated that in Co_3_(ndc)_3_(dabco), *Q_st_* rises from approximately 20 to 27 kJ·mol^−1^ with increasing CO_2_ loading [33].

Finally, the entropy change (ΔS) decreases progressively with increasing coverage, reflecting the restricted molecular mobility and enhanced ordering of CO_2_ within the CHA cages of H-SSZ-13. Similar behavior has been observed in other porous adsorbents: Gunawan et al. attributed negative *ΔS* values in zeolite-templated carbon to reduced molecular randomness [34], while Elsayed et al. reported negative *ΔS* values for CO_2_ adsorption in LTA and ITQ-29 zeolites, suggesting a more ordered configuration at the solid–gas interface [35]. Mechanistically, adsorption entropy reduction arises from the loss of translational and rotational degrees of freedom, and stronger confinement further amplifies this entropic penalty [36,37].

In addition to the qualitative agreement with adsorption theory, a quantitative comparison with literature data further supports the validity of the present results. Pham et al. [26] reported near-zero-coverage heats of CO_2_ adsorption for H-SSZ-13/6 and H-SSZ-13/12 of 35.2 and 26.8 kJ/mol, respectively, as summarized in their Table 2. In comparison, the enthalpy changes (*ΔH*) obtained in this work fall within a comparable energy range, particularly at higher relative uptake where |*ΔH*| approaches ~35 kJ·mol^−1^. It should be noted that the thermodynamic parameters in this work are derived using a different methodology. Specifically, the present platform utilizes the relative mass change detection capability of a resonant microcantilever to measure adsorption isotherms at multiple temperatures. These isotherms are then fitted using the Sips model, from which the thermodynamic parameters (*ΔG*, *ΔH*, and *ΔS*) are subsequently calculated. In contrast, the literature values are typically obtained from adsorption isotherms using virial or van’t Hoff analysis. Despite these differences in measurement principles and data processing methods, the overall agreement in energy scale indicates that the developed platform provides physically consistent thermodynamic measurements. It should also be noted that differences in Si/Al ratio, coverage definition, and parameter extraction methodology may lead to deviations in the exact numerical values; however, the consistency in energy scale and adsorption behavior confirms the reliability of the proposed system.

In summary, the variations in ΔG, *ΔH*, and *ΔS* observed in this work are consistent with theoretical expectations. Moreover, quantitative comparison with reported thermodynamic data for H-SSZ-13 demonstrates that the extracted energy scale is comparable to literature values, confirming that the developed system provides reproducible and physically meaningful thermodynamic data.

### 4.2. Advantages & Contributions

Compared with conventional adsorption instruments such as ASAP analyzers, microbalances, and infrared adsorption setups, the system developed in this study offers several advantages, including a miniaturized sensing module (97 × 75 × 22 mm, power consumption 1.2 W), high temporal resolution (10,000 data points per second), and direct applicability to real-time and in situ measurements. Beyond these engineering merits, this work also achieves methodological advancements in the extraction of adsorption thermodynamic parameters using resonant microcantilevers. Previous studies, such as that of Xu et al. (2014), modeled thermodynamic parameters by converting absolute frequency shifts into absolute adsorbed mass, followed by analysis using the Langmuir model to obtain ΔH, ΔG, and ΔS [11]. In contrast, the present system introduces several methodological improvements:

1. Relying on relative frequency shifts under equal relative uptake conditions, thus avoiding calibration errors caused by cantilever-to-cantilever variation and sample-deposition differences.
2. Employing the Sips model instead of the Langmuir model, enabling the characterization of heterogeneous adsorption sites and intermolecular interactions, while retaining the Langmuir model as a limiting case.
3. Adopting a stepwise concentration-gradient measurement protocol combined with on-chip rapid desorption enabled by an integrated micro-heater, and incorporates a fully automated LabVIEW-based testing software built on the Queued Message Handler (QMH) framework, thereby significantly reducing test duration and minimizing human-induced measurement errors.
4. Integrating multi-temperature isotherms for isosteric enthalpy fitting, which provides higher accuracy compared with conventional two-temperature methods.

Overall, these innovations establish the proposed system as a compact, fully integrated hardware–firmware–software platform capable of delivering more reliable and efficient thermodynamic measurements than both traditional adsorption instruments and previously reported cantilever-based approaches.

### 4.3. Limitations and Future Perspectives

Before outlining future research directions, it is important to clarify several limitations of the present study.

First, the current validation is restricted to single-component CO_2_ adsorption under low-pressure (ppm-level) conditions. This limitation arises from the strong adsorption affinity of H-SSZ-13 toward CO_2_, where higher concentrations would lead to rapid saturation and reduce the resolution of adsorption isotherms. While this constraint is material- and condition-dependent, the proposed methodology provides a useful framework that can be extended to higher concentration regimes with appropriate system adjustments.

Second, although the Sips model is employed to account for surface heterogeneity and intermolecular interactions, it may exhibit limitations at higher relative uptake, where the model tends to approach saturation behavior. This may reduce fitting accuracy in high-uptake regions and should be considered when interpreting thermodynamic parameters.

Third, the potential impact of sample deposition heterogeneity should also be considered. In principle, the proposed formulation eliminates the influence of the spring constant *k* through the definition of relative uptake, which improves robustness against variations in coating position and thickness. However, non-uniform sample distribution may still introduce secondary effects, such as localized mass loading or damping variations, which could affect measurement precision.

Building on the progress demonstrated in this study, several directions for future research can be identified. First, while the present system has been validated using H-SSZ-13 as a model adsorbent, extending this methodology to a wider range of materials—including other zeolites, metal–organic frameworks (MOFs), and hybrid adsorbents—will be an important next step. Further studies may also consider multicomponent gas mixtures and more complex environmental conditions to broaden the applicability of the platform.

Second, although the Sips model was employed here, the framework can be readily expanded to incorporate alternative adsorption models. These include the Tóth isotherm for heterogeneous surfaces [38], the Temkin model describing adsorbate–adsorbate interactions [39], and the Dubinin–Radushkevich equation for micropore filling [40], which together would enable a more comprehensive description of non-ideal adsorption behavior.

Third, the current implementation focuses on single-channel operation. Future development of multi-channel or high-throughput resonant cantilever arrays is expected to significantly enhance measurement efficiency while reducing experimental uncertainty, as demonstrated in recent studies on parallel adsorption analysis [41,42].

Overall, these considerations highlight both the current boundaries and the future potential of the proposed system, providing clear opportunities to further expand the capability, versatility, and impact of resonant cantilever-based adsorption thermodynamic measurement platforms.

## 5. Conclusions

In this study, a fully automated platform for gas–solid adsorption thermodynamic measurements was developed and systematically validated. The compact hardware–firmware–software integrated system demonstrates excellent stability, high temporal resolution, and ultra-low noise performance, enabling fast and precise control as well as real-time response over a wide temperature range. Using H-SSZ-13 as a representative adsorbent, the system successfully extracted key thermodynamic parameters (*ΔG*, *ΔH*, and *ΔS*) during CO_2_ adsorption, with results showing high reproducibility and strong consistency with adsorption theory and reported literature, thereby confirming the accuracy and physical significance of the proposed approach.

Compared with conventional adsorption characterization instruments, the developed system offers distinct advantages in miniaturization, real-time in situ measurement capability, and methodological innovation. The use of relative frequency-shift analysis under equal relative-uptake conditions, together with the incorporation of the Sips model, stepwise concentration-gradient protocols, and multi-temperature isotherm fitting, establishes an efficient and robust framework for adsorption thermodynamic analysis.

Overall, the proposed system demonstrates a flexible and scalable measurement framework. While the current study validates the approach using CO_2_ adsorption on a microporous zeolite (H-SSZ-13), its extension to other adsorbent materials, such as macroporous systems, chemisorption-dominated processes, and multicomponent gas mixtures, requires further experimental validation. Nevertheless, the generality of the hardware design and the adaptability of the analytical framework suggest strong potential for broader applications in gas–solid interface studies and adsorption thermodynamics.

## Figures and Tables

**Table 1:** Key performance metrics of the system, including frequency noise, temperature noise, PID temperature settling time, and frequency response settling time.

Data Sampling Rate (pts)	Frequency RMS Noise ^1^ (Hz)	Temperature RMS ^1^ Noise (°C)	PID Temperature Settling Time (0.1%) ^2^ (ms)	Frequency Response Settling Time (0.01%) (ms)
10	0.0083	0.0109	196.7	240
100	0.0093	0.0118	136	60
1000	0.0148	0.0119	31	17
10,000	0.0511	0.0464	24.9	9.6

^1^ Temperature RMS Noise was measured at 400 °C. Frequency RMS Noise was measured at room temperature. All parameters were measured in an N_2_ gas environment. ^2^ PID Temperature Settling Time refers to the step control from 50 °C to 400 °C. Frequency Response Settling Time refers to the settling time of frequency variation with temperature under the same step control (50 °C to 400 °C).

**Table 2:** Mean values and reproducibility of thermodynamic parameters (*ΔG*, *ΔH*, *ΔS*) for CO_2_ adsorption on H-SSZ-13 at 294.15 K (21 °C), evaluated at selected coverages.

Relative Uptake (%)	Gibbs Free Energy Change			Enthalpy Change			Entropy Change *	
	Mean (kJ·mol^−1^)	SD (kJ·mol^−1^)	RSD (%)	Mean (kJ·mol^−1^)	SD (kJ·mol^−1^)	RSD (%)	Mean (J·mol^−1^)	SD (J·mol^−1^)
0.4	−29.23	0.711	2.432	−10.011	1.212	12.107	65.335	4.648
0.8	−28.331	0.88	3.106	−13.037	1.461	11.207	51.991	5.346
1.2	−27.682	1.096	3.959	−16.808	1.82	10.828	36.967	6.294
1.6	−27.077	1.431	5.285	−22.683	2.287	10.082	14.938	7.629
2	−26.358	2.141	8.123	−35.299	2.419	6.853	−30.396	9.105

* RSD values are not reported for *ΔS* at certain coverages because the mean values approach zero.

## Data Availability

All data supporting the findings of this study are available within the article.
